# Workplace physical activity interventions and moderate-to-vigorous intensity physical activity levels among working-age women: a systematic review protocol

**DOI:** 10.1186/2046-4053-3-147

**Published:** 2014-12-19

**Authors:** Jennifer L Reed, Stephanie A Prince, Christie A Cole, J George Fodor, Swapnil Hiremath, Kerri-Anne Mullen, Heather E Tulloch, Erica Wright, Robert D Reid

**Affiliations:** Division of Prevention and Rehabilitation, University of Ottawa Heart Institute, 40 Ruskin Street, Ottawa, ON K1Y 4W7 Canada; Clinical Epidemiology Program, Ottawa Hospital Research Institute, Ottawa, ON K1Y 4E9 Canada; Health Sciences Library, University of Ottawa, Ottawa, ON K1H 8M5 Canada

**Keywords:** Motor activity, Women, Workplace, Occupation, Exercise, Physical activity, Systematic review

## Abstract

**Background:**

The rapid pace of modern life requires working-age women to juggle occupational, family and social demands. This modern lifestyle has been shown to have a detrimental effect on health, often associated with increased smoking and alcohol consumption, depression and cardiovascular disease risk factors. Despite the proven benefits of regular moderate-to-vigorous intensity physical activity (MVPA), few are meeting the current physical activity (PA) recommendations of 150 min of MVPA/week. It is important that appropriate and effective behavioural interventions targeting PA are developed and identified to improve the MVPA levels of working-age women. As these women spend a substantial proportion of their waking hours at work, workplaces may be an opportune, efficient and relatively controlled setting to implement programmes and strategies to target PA in an effort to improve MVPA levels and impact cardiometabolic health. The purposes of this systematic review are to compare the effectiveness of individual-level workplace interventions for increasing MVPA levels in working-age women in high-income/developed countries and examine the effectiveness of these interventions for improving the known beneficial health sequelae of MVPA.

**Methods/Design:**

Eight electronic databases will be searched to identify all prospective cohort and experimental studies that examine the impact of individual-level workplace interventions for increasing MVPA levels among working-age (mean age 18–65 years) women from high-income/developed countries. Grey literature including theses, dissertations and government reports will also be included. Study quality will be assessed using a modified Downs and Black checklist, and risk of bias will be assessed within and across all included studies using the Cochrane’s risk of bias tool and Grades of Recommendation, Assessment, Development and Evaluation approach. Meta-analyses will be conducted where possible among studies with sufficient homogeneity.

**Discussion:**

This review will determine the effectiveness of individual-level workplace interventions for increasing MVPA levels in working-age women in high-income/developed countries, and form a current, rigorous and reliable research base for policy makers and stakeholders to support the development and implementation of effective workplace interventions that increase MVPA levels in this population.

**Systematic review registration:**

PROSPERO CRD42014009704

## Background

The rapid pace of modern life requires working-age women to juggle occupational, family and social demands. On most days of the week, working-age women in North America which constitute 48% of the total workforce spend a substantial proportion of their waking hours at work, while also contributing more to unpaid work (e.g. cooking, cleaning, child care, gardening) as compared to their male counterparts [1, 2]. Further, women represent the largest proportion (79%) of single-parent families [3] and earn 22% to 33% less, on average, than males for equivalent full-time paid work [4–6]. Among lower income families, the need to work overtime or more than one job is also quite common [7], which leaves little time for women to prioritize their health. Indeed, it has been shown that women who work long hours exhibit higher rates of smoking and alcohol consumption and are more likely to be depressed [8].

According to the recent Canadian Health Measures Survey (CHMS) and National Health and Nutrition Examination Survey (NHANES) data, 28% to 31% of working-age women were classified as overweight, and 24% to 36% were classified as obese, respectively [9, 10]. An alarming proportion of working-age women in North America experience risk factors for cardiovascular diseases [11, 12], the leading cause of death in North America, including: high blood pressure (estimates of 19%–32%) [13, 14], high cholesterol (estimates of 11%–25%) [15, 16] and diabetes (estimates of 7%–11%) [17, 18]. Despite this, most women lack knowledge of cardiovascular disease risk factors, and substantial proportions (80%) are unaware of their own risk status [19].

Physical activity (PA) is an important modifiable health behaviour. Irrefutable evidence demonstrates the effectiveness of regular PA in the prevention of several chronic diseases including, but not limited to cardiovascular disease, high blood pressure, high cholesterol, diabetes, certain cancers and premature death [20–23]. The dose-response relationship is such that greater health benefits are achieved in proportion to increasing levels, within physiological limits, of PA [20–22]. According to the World Health Organization (WHO), adults should accumulate at least 150 min of moderate-to-vigorous intensity aerobic PA (MVPA) each week [24]. Examples of MVPA include brisk walking, running, cycling, lifting heavier loads, swimming and competitive sports. Most household activities are not vigorous enough to meet current MVPA recommendations [1, 25]. Unfortunately, despite the proven benefits of regular MVPA, very few (3%–14%) working-age women in North American, and less than the number of working-age men (4%–17%), are meeting current MVPA recommendations [23, 26]. Lack of time is one of the most commonly cited barriers to regular PA participation [27].

It is important that appropriate and effective behavioural interventions targeting PA are developed and identified to improve the MVPA levels of working-age women [28]. As this population spends a substantial proportion of their waking hours at work, workplaces may be an opportune, efficient and relatively controlled setting to implement programmes and strategies to target PA in an effort to improve MVPA levels and subsequently impact cardiometabolic health. Since employees with poor health and those with unhealthy lifestyles and chronic health conditions are less productive at work and take more sick leave [29–31], the potential to reduce absenteeism rates and healthcare costs may represent a strong incentive for the implementation of workplace programmes to increase MVPA levels to employers.

Although previous reviews have demonstrated the beneficial effects of workplace PA interventions on PA levels (i.e. minutes/hours per week), fitness, nutritional practices, body weight, psychosocial factors, work performance, health risks and healthcare cost outcomes among working-age adults [32–35], few have evaluated the impact on levels of MVPA [34, 35] and none have focused on working-age women from high-income Organization for Economic Co-operation and Development (OECD) countries [36] which exhibit poor adherence rates (≤50%) to current PA recommendations [23, 37]. The main objective of the proposed systematic review will be to compare the effectiveness of individual-level workplace interventions for increasing MVPA levels in working-age women in high-income/developed countries. The secondary objective will be to examine the effectiveness of these interventions for improving the known beneficial health sequelae of MVPA (e.g. weight, body mass index (BMI), body composition, waist circumference, blood pressure, blood serum lipids and glucose concentrations).

## Methods/Design

### Study design

A systematic review and meta-analysis will be performed to identify individual-level workplace interventions to increase MVPA levels in working-age women in high-income/developed countries. The systematic review will adhere to the reporting guidelines of the Preferred Reporting Items for Systematic Reviews and Meta-Analyses (PRISMA) statement [38] and will meet the items outlined in A Measurement Tool to Assess Systematic Reviews (AMSTAR) checklist [39, 40].

### Study registration

This systematic review is registered with PROSPERO (registration number: CRD42014009704; http://www.crd.york.ac.uk/PROSPERO).

### Types of participants

Studies will be included if the sample is largely comprised of working-age women (≥80% women in the sample or where female data can be extracted) from high-income/developed countries, defined according to OECD, with a mean age between 18 and 65 years.

### Types of exposures

All studies must contain an intervention component delivered in the workplace that is designed to increase MVPA levels. The interventions may include, but are not limited to: group aerobics classes; team sports; and walking, running or stair initiatives. The delivery of the interventions may be single- or multi-modal.

### Types of comparators

Since this systematic review will include all prospective cohort and experimental studies (randomized controlled trials (RCTs), pre-post design, quasi-experimental) studies that examine the impact of individual-level workplace interventions on increasing MVPA levels among working-age women, control groups will be used, when available, to compare effects. No restrictions will be placed on the control groups (e.g. no PA intervention, low intensity PA intervention).

### Types of outcomes

The primary outcome will be change in minutes per day of MVPA. MVPA is defined as a behaviour with an energy expenditure ≥3 metabolic equivalents (METs), ≥40% of VO_2_ reserve, ≥64% of peak heart rate, ≥12 rating of perceived exertion or >100 steps per minute [25, 41–44]. Measures of time (e.g. minutes per day) spent engaging in MVPA and where possible, a measure of variance around this outcome (e.g. standard error, 95% confidence intervals) will be extracted from all eligible and included studies regardless of the unit or method of MVPA measurement. MVPA can be either objectively measured (e.g. indirect calorimetry, accelerometers, pedometers, activity monitors) or self-reported (e.g. questionnaire, journal or log). Further, MVPA can be described using a composite measure of total time spent in MVPA or separately for moderate and vigorous intensities. Secondary outcomes including potential and known beneficial health sequelae of MVPA (e.g. weight, BMI, body composition, waist circumference, blood pressure, blood serum lipids, glucose concentrations) [20–22] will be extracted.

### Types of studies

We will include all prospective cohort and experimental (RCTs, pre-post design, quasi-experimental) studies that examine the impact of individual-level workplace interventions on increasing MVPA levels among working-age women from high-income/developed countries. Only articles available in English and French will be included as the authors are proficient in these languages. If there is an adequate number of RCTs, a summary of this evidence and the confidence in this evidence using the Grades of Recommendation, Assessment, Development and Evaluation (GRADE) approach [45] will be provided to increase internal validity of the systematic review. RCTs receive the highest grade with this approach.

### Search methods for the identification of studies

A comprehensive search strategy was designed in collaboration with a research librarian (EW), peer-reviewed by a second research librarian (SD), and includes a search of eight electronic databases: Ovid MEDLINE® In-Process and Other Non-Indexed Citations (1946 to present); EBM Reviews—Cochrane Database of Systematic Reviews (2005 to July 2014), EBM Reviews—Cochrane Central Register of Controlled Trials (1991 to present); EMBASE Classic + (1947 to present); CINAHL (1981 to present); Ovid PsycINFO (1806 to present); SPORTDiscus (1949 to present) and Dissertations and Theses (1980 to present). The strategy is illustrated using the MEDLINE search as an example (Table 1) and will be modified according to the indexing systems of the other databases. Grey literature (non-peer-reviewed works) that meets the inclusion criteria will be obtained including published lists of theses and dissertations, government reports and unpublished data and manuscripts (provided by original authors). Government reports will be searched using the Google search engine and a combination of key text words. Unpublished data and manuscripts will be solicited from original authors of studies that report on collecting MVPA. The bibliographies of all studies selected for the review will be examined to identify further studies as will those of previous reviews. The Google search engine will be used to identify studies that are published in non-indexed journals.

**Table 1:** **Sample MEDLINE search strategy**

Search terms	
**Workplace terms**	
1	Workplace/(13603)
2	workplace*.tw. (24754)
3	worksite*.tw. (2512)
4	(work* adj (place* or site* or location* or setting*)).tw. (5663)
5	“place of work”.tw. (656)
6	(employer* or employee* or worker*).tw. (158577)
7	(office adj2 work*).tw. (1799)
8	Occupational Health Services/(9650)
9	Occupational Health/(25082)
10	or/1-9 (201628)
**Physical activity terms**	
11	Motor Activity/(77808)
12	exp Exercise/(118458)
13	Physical Fitness/(21807)
14	“Physical Education and Training”/(11814)
15	exp Exercise Therapy/(29956)
16	Movement/(59273)
17	Bicycling/(7654)
18	Yoga/ (1506)
19	Accelerometry/(708)
20	(physical* adj (activit* or exercise* or fitness)).tw. (71697)
21	((fitness or exercise) adj (class* or course* or program* or training)).tw. (17628)
22	(“aerobic exercis*” or aerobics).tw (5489)
23	(walk*.tw or run*.tw or bike.tw, bicycl*.tw).tw (81389)
24	yoga.tw. (2019)
25	(pedomet* or acceleromet*).tw. (8153)
26	((moderate or high or vigorous) adj “intensity activit*”).tw (444)
27	(moderate-vigorous adj2 activit*).tw (166)
28	(“moderate to vigorous” adj2 activit*).tw (1852)
29	MVPA.tw. (1267)
30	or/11-29 (380469)
31	10 and 30 (5432)
**Publication type terms**	
32	Intervention Studies/(6743)
33	intervention*.tw. (552005)
34	Program Evaluation/(45950)
35	evaluation studies/(194196)
36	Multicenter Study/(173099)
37	Observational Study/(2632)
38	(observational adj (study or studies)).tw. (45553)
39	Randomized Controlled Trials as Topic/(93384)
40	randomized controlled trial/(375396)
41	randomi?ed.tw. (377695)
42	exp Clinical Trials as Topic/(281076)
43	clinical trial/(488142)
44	controlled clinical trial/(88473)
45	(clinical adj trial*).tw. (216982)
46	case–control studies/(182562)
47	exp Cohort Studies/(1353453)
48	Meta-Analysis/(48552)
49	(meta-analysis or metaanalysis).tw. (54480)
50	“review”/(1882177)
51	systematic review.tw. (47622)
**Combining search terms**	
52	or/32-51 (4551591)
53	31 and 52 (2403)

### Selection of studies

Articles will be imported into Microsoft Excel (Microsoft Canada Inc. Mississauga, ON, Canada), and all duplicates will be removed; only the most relevant article per data source/analysis will be retained. Two independent reviewers (JLR, CAC) will screen the titles and abstracts of all articles to identify potentially relevant articles. Full texts of each potentially relevant article identified by either reviewer during the title and abstract screening phase will be reviewed to determine whether the title and abstract screening inclusion criteria are met. The full texts of all potential articles that meet the inclusion criteria will be obtained and reviewed. Two independent reviewers will screen the full texts for inclusion (JLR, CAC). Any disagreements between the reviewers will be resolved by consensus and or discussion with a third reviewer (SAP). Intra-class correlations will be calculated to assess agreement between the reviewers. Reviewers will not be blinded to the authors or journals when screening articles.

### Data collection

Prior to data extraction, a data extraction form will be created and tested by the research team using a subset of the included studies. The extraction form will be modified based on feedback from the research team to improve its usability and ensure that complete and pertinent data is obtained. Standardized data abstraction forms including quality assessments will be completed by both reviewers (JLR and CAC). Any disagreements will be resolved by consensus and or discussion with a third reviewer (SAP or RDR). Reviewers will not be blinded to the authors or journals when extracting data.

From each prospective cohort and experimental study, the following data will be extracted: publication details (authors, year, country of study), participants’ characteristics (age range, mean age, sex distribution, chronic diseases, health states, population, setting), sample size, study design (RCT, pre-post, quasi-experimental), time points when data were collected (e.g. 3 weeks, 4 months), length of follow-up, intervention details, description of control, usual care or wait list-control group, information regarding blinding and randomization techniques, MVPA measurement method and whether self-report or objective tools were used, MVPA units of measurement, statistical analyses methods (i.e. *t*-tests, linear modeling), effect of the intervention on MVPA (effect size, 95% CI, standard mean error or deviation) and effect of intervention on known beneficial health sequelae of MVPA (weight, BMI, body composition, waist circumference, blood pressure, blood serum lipids, glucose concentrations) [20–22]. In cases where several publications report the same results from the same data source, only one article per data source/analysis will be retained to avoid double counting. If an investigator uses a measure that has the potential to capture MVPA (e.g. FITT log, accelerometers) but does not report on these outcomes in the manuscript, or if a paper reports on a study protocol, the authors will be contacted to determine whether the MVPA results can be obtained; other missing data to determine inclusion criteria (e.g. study design, age distribution, sex distribution) will also be obtained. A maximum of two e-mail or phone call attempts will be made to contact the corresponding author of these articles to obtain additional data.

### Quality and risk of bias within studies

The Downs and Black checklist will be used to assess the quality and risk of bias of the individual studies [46]. The checklist contains 27 items, with a maximum possible score of 32 points [46]. We will simplify the scoring of item 27 from a five-point range to a binary system, granting one point (1) for adequate power calculations or no points (0) if power was not adequately addressed. The maximum possible score for the modified checklist will be 28 points with higher scores indicating superior quality. The quality of the individual studies will be rated by reviewer CAC and verified by reviewer JLR. The quality scores will be used for performing subgroup analyses (high-quality vs. low-quality). The Cochrane Collaboration’s tool will be used to assess risk of bias for each RCT. Items included in Cochrane’s risk of bias assessment include: sequence generation (randomization); allocation concealment; blinding of participants, personnel and investigator; incomplete data (e.g. losses to follow-up, intention-to-treat analysis); selective outcome reporting; and other possible sources of bias. The risk of bias assessment will be carried out by two independent assessors (JLR and CAC); any disagreements between assessors will be resolved by consensus and or through discussion with a third reviewer (SAP).

### Quality of the evidence

The quality of the evidence for the RCTs will be assessed as high, moderate, low or very low using the GRADE approach [45]. With the GRADE approach, the highest quality rating is for RCT evidence. In addition to study design, the quality of evidence will be rated upon possible risk of bias, imprecision, heterogeneity, indirectness or suspicion of publication bias. Risk of bias for the RCTs will be assessed using Review Manager (RevMan) 5.3.3 (The Nordic Cochrane Centre, The Cochrane Collaboration, 2012) [47] and then imported into GRADEprofiler (GRADEpro) Version 3.6.1 [48] to create a summary of findings table and rate the quality of the evidence using the GRADE approach.

### Analysis

Forest plots and meta-analyses will be created using RevMan 5.3.3 to synthesize the measures of effect (e.g. mean differences) and 95% confidence intervals for each intervention on MVPA. Forest plots and meta-analyses will only be performed when the included studies are sufficiently homogenous in terms of study design, participants, interventions and outcomes to provide a meaningful summary measures. A random-effects meta-analysis will be used as effect sizes are likely to be similar, but not identical across all studies. Inverse variance methods will be used for continuous data and DerSimonian Laird methods for dichotomous data. Heterogeneity will be assessed using the *I*^2^ statistic with values above 75% and *p* < 0.10 used to indicate high heterogeneity across studies [49]. If high heterogeneity is found, a meta-analysis will not be performed. A funnel plot of the included studies’ estimates of effect will be used to assess the presence of publication bias. Funnel plots will only be performed if ten or more studies are included. The plots will be assessed both visually and by using Egger’s test, with *p* < 0.10 used to indicate the presence of a significant publication bias [50].

### Subgroup analyses

Several subgroup analyses will be performed if sufficient data are available. These analyses will examine differences between: age (e.g. 18–24 years vs. 25–44 years vs. 45–65 years); number of children; education (e.g. high school vs. post-secondary vs. graduate); marital status (e.g. married vs. unmarried); occupation (e.g. active vs. sedentary jobs); worksite (e.g. office vs. hospital); working status (e.g. casual, part-time [<37.5 h/week] vs. full-time [37.5–40 h/week] vs. excessive overtime [>40 h/week]); income; self-reported and objectively measured MVPA; intervention focus (e.g. walking vs. aerobic classes vs. team sports vs. exercise and diet programmes vs. gym membership), intervention mode (e.g. web-based vs. paper-based); intervention delivery (e.g. single- vs. multi-modal); study design (e.g. control group vs. no control group, randomized vs. non-randomized controlled trial); control groups (e.g. no PA intervention vs. low intensity PA intervention) and impact on known beneficial health sequelae of MVPA (e.g. weight vs. BMI vs. body composition vs. waist circumference vs. blood pressure vs. blood serum lipids vs. glucose concentrations). Subgroup analyses will be used to explore heterogeneity, in addition to any clinical interest in the differences between groups.

## Discussion

This systematic review will be the first, to our knowledge, to determine the effectiveness of individual-level workplace interventions for increasing MVPA levels in working-age women in high-income/developed countries. The findings from this review will provide a current, rigorous and reliable research base for policy makers and stakeholders to support the design and implementation of effective workplace interventions that increase MVPA levels in working-age women in high-income/developed countries that likely have poor health and accumulate low MVPA levels. The findings from this review will be disseminated for scientific peer-reviewed open access publication, as well as conference presentation and proceedings. The review authors will also disseminate the findings to health researchers and academic institutions through national and international seminars and workshops.

## Abbreviations

MVPA: Moderate-to-vigorous intensity physical activity
PA: Physical activity
CHMS: Canadian Health Measures Survey
NHANES: National Health and Nutrition Examination Survey
WHO: World Health Organization
OECD: Organization for Economic Co-operation and Development
BMI: Body mass index
PRISMA: Preferred Reporting Items for Systematic Reviews and Meta-Analyses
AMSTAR: A Measurement Tool to Assess Systematic Reviews
RCT: Randomized controlled trials
METS: Metabolic equivalents
GRADE: Grades of Recommendation, Assessment, Development and Evaluation.
